# L-Arginine Enhances Intracellular Killing of Carbapenem-Resistant *Klebsiella pneumoniae* ST258 by Murine Neutrophils

**DOI:** 10.3389/fcimb.2020.571771

**Published:** 2020-11-13

**Authors:** Hernán F. Peñaloza, Danielle Ahn, Bárbara M. Schultz, Alejandro Piña-Iturbe, Liliana A. González, Susan M. Bueno

**Affiliations:** ^1^ Millennium Institute on Immunology and Immunotherapy, Departamento de Genética Molecular y Microbiología, Facultad de Ciencias Biológicas, Pontificia Universidad Católica de Chile, Santiago, Chile; ^2^ Department of Pediatrics, Columbia University Medical Center, New York, NY, United States

**Keywords:** *Klebsiella pneumoniae* ST258, neutrophils, phagocytosis, phagosome acidification, l-arginine

## Abstract

Carbapenem-resistant *Klebsiella pneumoniae* ST258 (CRKP-ST258) are a global concern due to their rapid dissemination, high lethality, antibiotic resistance and resistance to components of the immune response, such as neutrophils. Neutrophils are major host mediators, able to kill well-studied and antibiotic-sensitive laboratory reference strains of *K. pneumoniae*. However, CRKP-ST258 are able to evade neutrophil phagocytic killing, persisting longer in the host despite robust neutrophil recruitment. Here, we show that neutrophils are unable to clear a CRKP-ST258 isolate (KP35). Compared to the response elicited by a prototypic *K. pneumoniae* ATCC 43816 (KPPR1), the neutrophil intracellular response against KP35 is characterized by equivalent production of reactive oxygen species (ROS) and myeloperoxidase content, but impaired phagosomal acidification. Our results ruled out that this phenomenon is due to a phagocytosis defect, as we observed similar efficiency of phagocytosis by neutrophils infected with KP35 or KPPR1. Genomic analysis of the *cps* loci of KPPR1 and KP35 suggest that the capsule composition of KP35 explain the high phagocytosis efficiency by neutrophils. Consistent with other reports, we show that KP35 did not induce DNA release by neutrophils and KPPR1 only induced it at 3 h, when most of the bacteria have already been cleared. l-arginine metabolism has been identified as an important modulator of the host immune response and positively regulate T cells, macrophages and neutrophils in response to microbes. Our data show that l-arginine supplementation improved phagosome acidification, increased ROS production and enhanced nitric oxide consumption by neutrophils in response to KP35. The enhanced intracellular response observed after l-arginine supplementation ultimately improved KP35 clearance *in vitro*. KP35 was able to dysregulate the intracellular microbicidal machinery of neutrophils to survive in the intracellular environment. This process, however, can be reversed after l-arginine supplementation.

## Introduction

*Klebsiella pneumoniae* (KP) is an extremely versatile bacterium able to colonize different environments including different kinds of soil, water courses, medical devices and mucosal surfaces (Tzouvelekis et al., 2012; Paczosa and Mecsas, 2016). KP genetic flexibility is mainly due to its increased ability to acquire DNA from the environment or nearby bacteria (Gomez-Simmonds and Uhlemann, 2017) and is likely a reason that KP has easily become resistant to common antibiotics and is now a major health concern worldwide. In the last 30 years, KP has had a major epidemiologic impact due to the emergence and dissemination of antibiotic multi-resistant strains and hypervirulent strains (Tzouvelekis et al., 2012; Patel et al., 2014; Paczosa and Mecsas, 2016; Gomez-Simmonds and Uhlemann, 2017; Gu et al., 2018). Among multi-drug resistant KP, carbapenem-resistant sequence type 258 (CRKP-ST258) have been described as the most successful sequence type due to its rapid worldwide dissemination and lethally (Munoz-Price et al., 2013).

Compared to classical antibiotic susceptible KP, CRKP-ST258 show reduced virulence, lower LD_50_ (Tzouvelekis et al., 2013), and higher susceptibility to serum or antibody-mediated killing (Olonisakin et al., 2016; DeLeo et al., 2017) in *in vivo* models of infection. However, CRKP-ST258 have acquired resistance to neutrophil phagocytic killing (Kobayashi et al., 2016) that can be reversed by the supportive action of anti-capsular antibodies (Kobayashi et al., 2018). The evasion of neutrophil killing by pathogenic bacteria can be achieved by several mechanisms and is not restricted to impaired phagocytosis. It can involve the induction of neutrophil death as well as the intracellular bacterial survival (Urban et al., 2006). Pathogenic bacteria use several strategies to survive neutrophilic intracellular killing, including the inhibition of phagosome maturation, survival within the phagosome or escape to the cytoplasm (Urban et al., 2006). The main mechanism that KP use to avoid being killed by neutrophils is evasion of phagocytosis (Kobayashi et al., 2016), mostly driven by the composition of the polysaccharide capsule (Paczosa and Mecsas, 2016). A recent study reported considerable heterogeneity in capsule composition among CRKP-ST258 isolates due to mutations in capsule-related genes located in the capsular polysaccharide synthesis (*cps*) locus (Ernst et al., 2020); and a different study described the ability of phagocytosed KP to survive within the macrophage-phagosome by preventing the maturation of the phagolysosome (Cano et al., 2015). These two studies suggest that the ability of CRKP-ST258 to evade phagocytosis by neutrophils is not restricted to an uptake defect, and these bacteria potentially survive intracellularly by disrupting phagosomal machinery in these immune cells.

In a previous report, using a representative CRKP-ST258 isolate from New York City (KP35) (Gomez-Simmonds et al., 2015), we characterized the immune response evoked in a murine model of pneumonia. Although neutrophils are rapidly recruited to the lungs, KP35 survive in the lung tissue for at least 10 days post infection (Ahn et al., 2016; Peñaloza et al., 2019b). Along with neutrophils, monocytic myeloid-derived suppressor cells (M-MDSCs) were also rapidly recruited during KP35 pneumonia (Ahn et al., 2016), and the production of IL-10 by these cells promotes a balanced immune response that ultimately aids in the clearance of KP35 from the airways (Peñaloza et al., 2019b). These cells suppress the inflammatory response through different mechanisms, including production of IL-10 and depleting l-arginine through the activity of Arginase-1 and iNOS (Peñaloza et al., 2019a). The role of l-arginine in neutrophil function is controversial: whereas one report showed that l-arginine supplementation improves *Staphylococcus aureus* phagocytosis (Moffat et al., 1996), another study showed that l-arginine supplementation does not affect the neutrophilic response against *Aspergillus fumigatus* (Kapp et al., 2014).

In the present report we provide evidence that KP35, a representative clinical isolate of CRKP-ST258, evade the neutrophil intracellular response by preventing the normal activity of the intracellular machinery after phagocytosis, a process that can be reversed through the supplementation of l-arginine.

## Materials and Methods

### Ethics Approval

All experimental protocols were reviewed and approved by the Scientific Ethical Committee for Animal and Environment Care (Comité Ético Científico para el Cuidado de Animales y Ambiente) of the Pontificia Universidad Católica de Chile (protocol numbers 150721004 and 150721005).

### Murine Neutrophil Isolation and Killing Assay

Bone marrow (BM) from femurs and tibias of C57BL6/J mice were flushed with RPMI 1640 medium supplemented with 10% FBS and EDTA 2 mM (US Biological E2210). Erythrocytes were osmotically lysed using a solution of NaCl 0.2% and NaCl 1.6%. Once BM cells were isolated, neutrophils were purified by magnetic negative neutrophil isolation kit for mouse (MACS Miltenyi Biotec) according to manufacturer instructions. Next, neutrophils were counted, and resuspended in HBSS containing Ca^2+^ and Mg^2+^ supplemented with 0.1% gelatin (Sigma- Aldrich G7765). To evaluate bacterial killing, neutrophils (1 × 10^5^ cells/200 µl) were incubated with opsonized KPPR1 or KP35 (MOI = 1) for 120 min with slow rotation at 37°C. After 120 min, each tube was placed at 4°C to stop the reaction and the total viable cells were counted in a conventional hemocytometer with trypan-blue exclusion. Cells were lysed with 1% saponin (Sigma-Aldrich 47036) and bacterial viability was determined by serial dilutions.

### Neutrophil Phagocytosis by Differential Centrifugation

BM Neutrophils (1 × 10^5^ cells/200 µl) were infected with opsonized KPPR1 and KP35 (MOI = 1) for 10 or 30 min with slow rotation at 37°C. After 10 or 30 min each tube was placed at 4°C. Then, to separate extracellular and intracellular bacteria, we followed a two-step colony forming assay (Parker et al., 2014). Briefly, each sample was centrifuged at 100 g for 5 min at 4°C. Then the supernatant was collected and saved, avoiding the disturbance of the neutrophil pellet. Cells were washed twice with ice-cold sterile 1× PBS and resuspended in sterile 1× PBS. Finally, neutrophils were lysed with 1% saponin (Sigma-Aldrich 47036) and bacterial viability in pellet lysates and supernatant were determined by plating serial dilutions on LB agar.

### Neutrophil Phagocytosis by Flow Cytometry

KPPR1 and KP35 were stained with FITC (0.25 mg/ml) in staining buffer (50 mM NaHCO_3_, 342 mM NaCl, in ddH_2_O) for 30 min on ice. Then, neutrophils (1 × 10^5^ cells/200 µl) were infected with FITC labeled KPPR1 or KP35 (MOIs = 10 and 50) in rotation at 37°C, 5% CO_2_ for 10 min. Samples were fixed with 2% PFA and analyzed in a BD FACSCanto II at Columbia University Medical Center. Trypan blue 0.4% was added to each sample to quench extracellular FITC fluorescence.

### Transmission Electron Microscopy (TEM)

5 × 10^5^ isolated BM neutrophils were resuspended in HBSS Ca^2+^ Mg^2+^ with 0.1% gelatin and infected with KP35 or KPPR1 (MOI = 10) for 30 min in slow rotation at 37°C, 5% CO_2_. At 30 min, neutrophils were centrifuged at 1,400 rpm for 7 min and fixed with 2.5% glutaraldehyde in 0.1 M sodium cacodylate buffer (pH = 7.2). Once fixed, samples were prepared and analyzed in a Philips Tecnai 12 (Biotwin) Transmission microscope with the help of the Advanced Microscopy Unit of Pontificia Universidad Católica of Chile.

### Comparison of the *cps* Loci From Strains NJST258_1, KP35, and KPPR1

The genomes of KP NJST258_1 and KPPR1 (Accession numbers: NZ_CP006923.1 and NZ_CP009208.1), and the contigs containing the *cps* locus from KP35 (Accession numbers: LRXK01000026.1, LRXK01000031.1 y LRXK01000117.1) were obtained from GenBank. KP35 contigs were ordered in Mauve v.2015026 build 10 (Rissman et al., 2009) and annotated using RAST (https://rast.nmpdr.org/) (Aziz et al., 2008; Overbeek et al., 2014; Brettin et al., 2015). The capsular type and annotations of some *cps* genes were determined using Kaptive (https://kaptive-web.erc.monash.edu/) (Wick et al., 2018). Finally, the tBLASTx comparisons and the corresponding figure were made in EasyFig v.2.2.2 (Sullivan et al., 2011) using BLAST+ v.2.9.0.

### Extracellular DNA Quantification

BM neutrophils (1 × 10^5^/300 µl) were stimulated with KPPR1 and KP35 (MOIs= 10, 25 or 50) for 10 and 180 min at 37°C with 5% CO_2_. Then, culture supernatant was collected, and extracellular DNA was measured using Quant-iT PicoGreen dsDNA (Invitrogen), following the manufacturer’s instructions.

### Phagosome Acidification Assay

BM neutrophils were resuspended in HBSS Ca^2+^ Mg^2+^ and then separated in three different groups: neutrophils incubated with LysoTracker Red DND-99 (50 nM) (Invitrogen L7528); neutrophils incubated with LysoTracker Red DND-99, with the acidification inhibitor chloroquine diphosphate (100 µM) (Sigma-Aldrich C6628); and neutrophils alone. Cells were incubated for 30 min at 37°C, 5% CO_2_. After incubation, 1 × 10^5^ Neutrophils were infected with KP35 or KPPR1 (MOI = 10) in a final volume of 200 µl for 60 min at slow rotation at 37°C, 5% CO_2_. After the time of infection, neutrophils were fixed with 500 µl of 2% paraformaldehyde at room temperature, in darkness, for 15 min. After fixation, cells were washed twice with sterile 1× PBS and acidification fluorescence was read in the flow cytometer in the PE channel (blue laser 488 nm, 575/25 filter). Analyses were performed in a BD LSR FORTESSA X-20 at the Pontificia Universidad Católica de Chile. All data were analyzed using FlowJo vX.0.7

### Neutrophil L-Arginine Supplementation

Murine neutrophils were supplemented with 200 mg/L of l-arginine (Sigma-Aldrich W381918) for 30 min prior KP infection. KP35 killing and phagosome acidification were evaluated as described above. To evaluate a possible impact on KP35 growth in presence of l-arginine, KP35 was growth in a nutrient rich medium (LB) and a nutrient poor medium (HBSS) non-supplemented and supplemented with l-arginine (100 mg/L and 200 mg/L), and OD_600_ was measured every 20 min during 160 min.

### ROS Production by Neutrophils

BM neutrophils (1 × 10^5^ cells) were incubated in 96-well black plate treated with 0.01% poly-l-lysine (Sigma Aldrich P4707) in presence of the general oxidative stress indicator Carboxy-H2DCFDA (Thermo Fisher C400), 10 µM, for 60 min at 37°C. Then, neutrophils were washed to remove excess probe and infected with opsonized KPPR1, KP35 and *Staphylococcus aureus* USA300 (positive control) (MOI = 1) and centrifuged at 524g for 8 min at 4°C to synchronize phagocytosis (Kobayashi et al., 2016). After centrifugation, the plate was kept in a plate reader at 37°C. ROS production was evaluated every 10 min for a total time of 80 min at Ex = 495/Em = 527 nm. ROS production in l-arginine-supplemented neutrophils was evaluated every 4 min during 32 min.

### Nitric Oxide Production by Neutrophils

BM Neutrophils (1 × 10^5^ cells) were incubated in 96-well black plate treated with 0.01% poly-l-lysine (Sigma Aldrich P4707) in presence of the NO substrate DAF-FM Diacetate (Thermo Fisher D23844), 10 µM, for 60 min at 37°C. Next, neutrophils were washed to remove excess probe and incubated for 30 min. Then, cells were infected with opsonized KPPR1, KP35 and *S. aureus* USA300 (MOI = 1) and centrifuged at 524 g for 8 min at 4°C to synchronize phagocytosis (Kobayashi et al., 2016). After centrifugation, the plate was kept it in a plate reader at 37°C. NO production was evaluated every 3 min during 30 min at Ex = 495/Em = 515 nm.

### MPO Production

5 × 10^5^ isolated BM neutrophils were resuspended in HBSS Ca^2+^ Mg^2+^ with 0.1% gelatin and infected with KP35 or KPPR1 (MOI = 1) for 30 min in slow rotation at 37°C. Cell culture supernatant was then collected after spinning at 1200 rpm for 10 min. The myeloperoxidase content was then measured using the Neutrophil Myeloperoxidase Activity Assay Kit (Cayman Chemical) according to the manufacturer’s instructions.

### Cytokine Production by Infected Neutrophils

Supernatants from uninfected neutrophils, neutrophils infected with KPPR1 and neutrophils infected with KP35 were collected after 30 min and 60 min post infection. Production of neutrophil-relevant cytokines such as G-CSF, IL-1α, IL-1β, IL-10, TNF-α, and IL-12p70 was quantified using a 31-plex mouse discovery assay (Eve Technologies).

### Statistical Analyses

Single comparisons of bacterial killing at 2 h and phagocytosis at 10 and 30 min by differential centrifugation were analyzed using a Student’s t-test. Neutrophil survival, phagosome acidification of neutrophils infected with KP35 and KPPR1; KP35 killing after l-arginine supplementation, ROS and NO production, and KP35 growth in the presence and absence of l-Arginine were compared with a two-way ANOVA test followed by a Tukey’s test for multiple comparisons. KP phagocytosis by flow cytometry, DNA release, phagosome acidification in l-arginine supplemented neutrophils, MPO production and cytokine production were compared through a one-way ANOVA test followed by a Tukey’s test for multiple comparisons. All comparisons were performed using the GraphPad PRISM 8.2.0 software for Macintosh.

## Results

### Phagocytosis and Extracellular Response by Neutrophils in Response to KP35 and KPPR1

**Figure 1:**
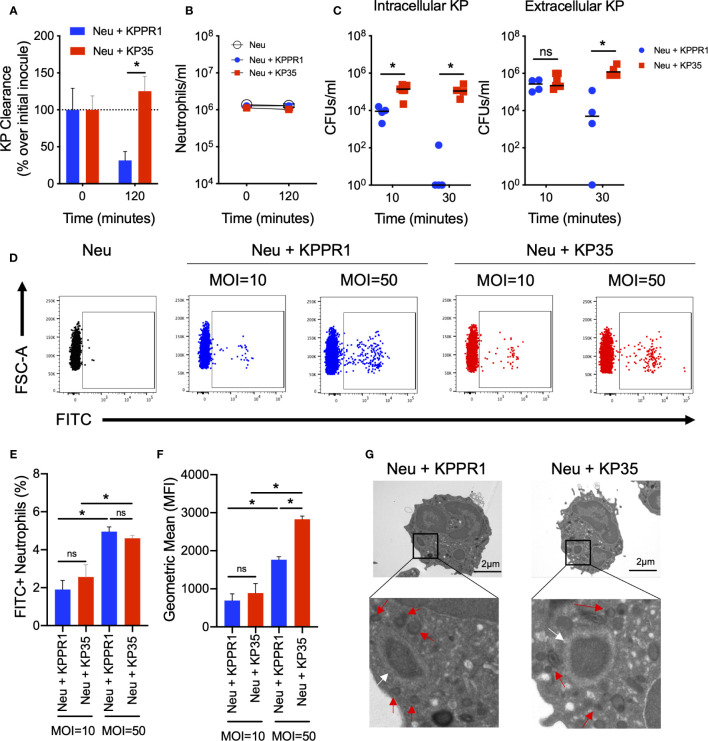
KP35 evades neutrophil killing but does not prevent phagocytosis. **(A)** KP35 and KPPR1 killing (MOI = 1) and **(B)** neutrophil count were evaluated for 2h. **(C)** Neutrophil phagocytosis of KP35 and KPPR1 (MOI = 1) were quantified by differential centrifugation in LB agar plates after 10 and 30 min. **(D–F)** These results were confirmed by flow cytometry (MOIs = 10 and 50) after 10 min. **(G)** KPPR1-phagosome and KP35-phagosome were visualized by TEM after 30 min (white arrows) and granules surrounding the phagosome (red arrows) were identified from the pictures obtained. **(A–C)**, *P < 0.05, for single comparisons (t-test). **(D–F)**, *P < 0.05, for multiple comparisons, one-way ANOVA test followed by a Tukey’s multiple test comparison. ns, non significant.

We first evaluated whether the resistance of KP35 to neutrophil killing was due to the inability of neutrophils to phagocytose the bacterium. We evaluated neutrophil phagocytosis of two different KP strains, KPPR1 (*K. pneumoniae* ATCC 43816), a classical KP strain that is easily killed by neutrophils (**Figure 1A**) and KP35, a clinical CRKP-ST258 isolate that is resistant to neutrophil killing (**Figure 1A**). None of these strains induced neutrophil death in the first 2 h of infection (**Figure 1B**), although 4 h post infection, KP infected-neutrophil viability rapidly decreased compared with uninfected neutrophils (**Supplementary Figure 1**). Ingestion of KP35 and KPPR1 by neutrophils was evaluated at 10 and 30 min by differential centrifugation. Our data show that KPPR1 is rapidly ingested and killed by neutrophils after 10 and 30 min (**Figure 1C**). At 10 min, we measured less intracellular KPPR1 suggesting rapid uptake and killing of the bacteria, with reduction of both extracellular and intracellular bacteria at 30 min. This finding was not observed with KP35, which had a relatively stable intracellular and extracellular bacterial burden at 10 and 30 min (**Figure 1C**). Given that there was reduced intracellular burden of KPPR1 as compared with KP35, we evaluated KP phagocytosis by neutrophils by flow cytometry, using FITC-labeled bacteria (**Figure 1D**). After 10 min, the percentage of FITC+ neutrophils were equivalent when neutrophils were infected with KPPR1 and KP35; this was MOI-dependent, with greater uptake of bacteria when the neutrophils were infected with a MOI of 50 (**Figure 1E**). Consistent with the findings obtained by differential centrifugation, KP35-infected neutrophils presented a higher FITC geometric mean fluorescence intensity (MFI) compared to KPPR1-infected neutrophils at MOI = 50, indicating that while KPPR1 is being rapidly phagocytosed and cleared, KP35 survives inside neutrophils after ingestion (**Figure 1F**).

We next visualized the phagocytosed bacteria and KP-containing phagosomes in KPPR1- and KP35-infected neutrophils by transmission electron microscopy (TEM) (**Figure 1G**). Finally, we evaluated whether the phagocytic response against KPPR1 and KP35 induced the production of cytokines by neutrophils. After 30 and 60 min, low levels of G-CSF, IL-1α, IL-1β, and IL-12p70 were detected (**Supplementary Figure 2**). At 60 min, TNF-α and IL-10 were poorly produced but showed an upward trend in neutrophils infected with KPPR1 and KP35 respectively (**Supplementary Figures 2J, L**).

**Figure 2:**
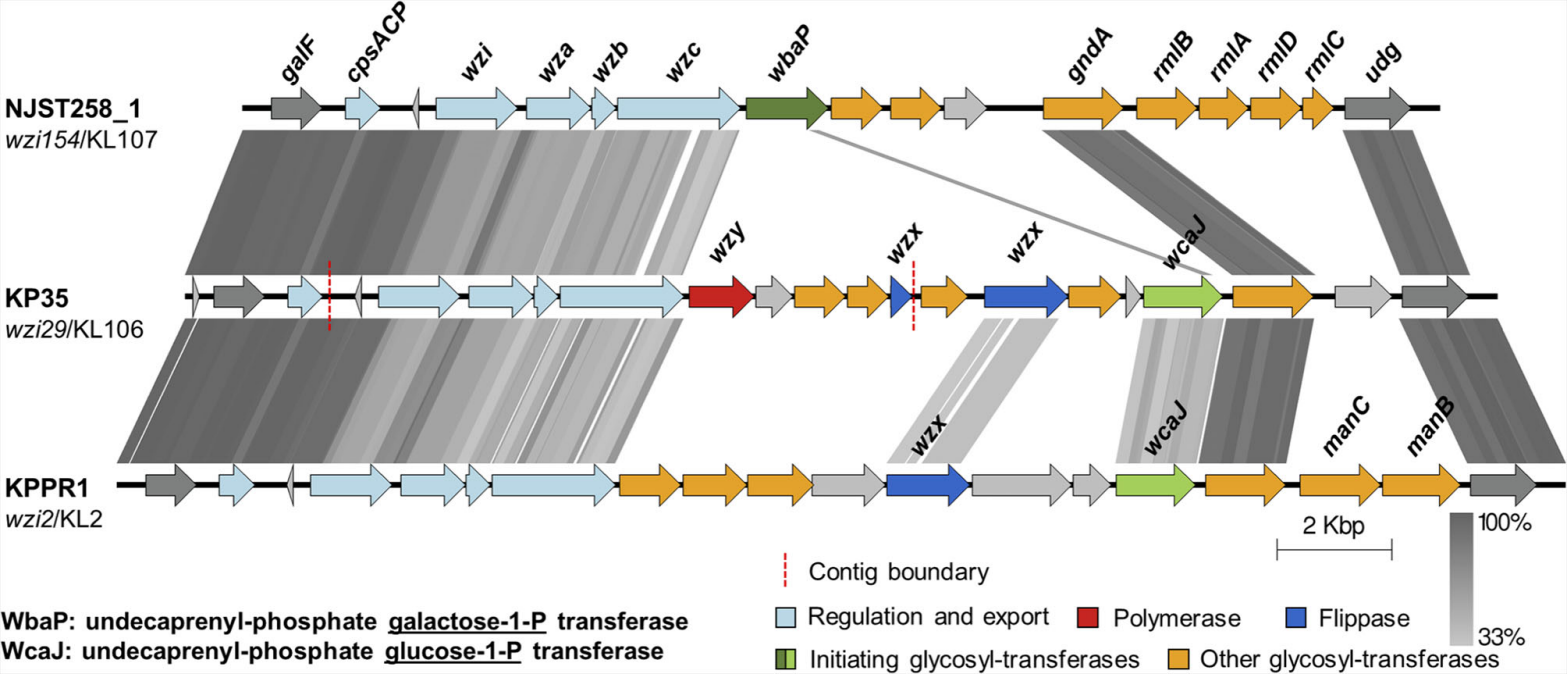
The capsular polysaccharide synthesis (*cps)* loci of KP35 and KPPR1 encode the glycosyl-transferase initiator WcaJ. tBLASTx comparisons of the *cps* loci found in strains NJST258_1, KP35 and KPPR1. Grey lines represent identity at the amino acid level, from 33 to 100%. The *wzi* allele and capsular type are indicated below each strain name. The regions showed in the figure correspond to coordinates: 1793433 to 1814332, for NJST258-1 (NZ_CP006923.1); 274446 to 297347 for the contigs of KP35 (see methods); and 5170058 to 5195339 for KPPR1 (NZ_CP009208.1).

The polysaccharide capsule is the main virulence factor that confers KP resistance to phagocytosis. The sequence comparison between the *cps* loci of KPPR1, KP35 and NJST258_1, a CRKP-ST258 isolate resistant to neutrophil phagocytosis (Kobayashi et al., 2016), showed the expected locus structure characterized by the conserved genes in the terminal regions (*galF*, *cpsACP*, *wzi, wza, wzb*, *wzc*, *gnd*, and *udg*), which encode the core proteins involved in regulation, synthesis and export; and the central variable region that contains genes encoding capsule-specific glycosyl-transferases and the core assembly proteins Wzx and Wzy (**Figure 2**). Analysis of the *cps* region of KP35 revealed that this strain carries a clade I *cps* locus (*wzi29*/KL106) that encodes a different repertoire of glycosyl-transferases compared with NJST258_1 (clade II *cps* locus *wzi154*/KL107), including the glycosyl-transferase responsible for initiating the capsule biosynthesis. Whereas KP35 harbors *wcaJ* — which encodes an undecaprenyl-phosphate glucose-1-P transferase — NJST258_1 lacks this gene and carries *wbaP*, encoding an undecaprenyl-phosphate galactose-1-P transferase. Interestingly, KPPR1, which is phagocytosed by neutrophils as well as KP35, also harbors the *wcaJ* gene (**Figure 2**). These findings suggest that the ingestion of KP35 by neutrophils may be allowed due to its capsule composition.

**Figure 3:**
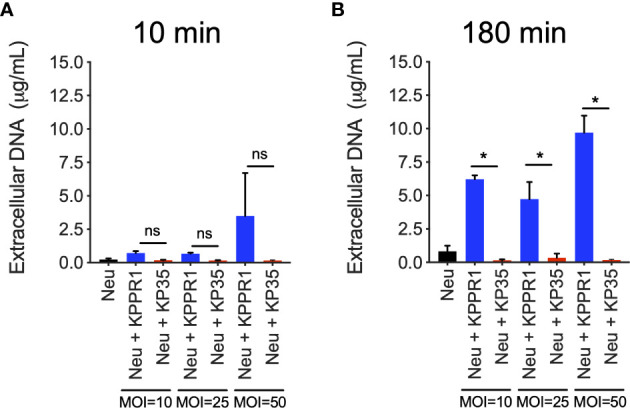
KP35 does not induce release of neutrophil DNA to the extracellular space. DNA release to the supernatant by KPPR1 and KP35 (MOIs 10, 25 and 50) infected neutrophils was evaluated at **(A)** 10 min and **(B)** 180 min. *P < 0.05, for multiple comparisons, one-way ANOVA test followed by a Tukey’s test for multiple comparisons. ns, non significant.

We next evaluated the extracellular response to KPPR1 and KP35 by neutrophils *in vitro*. We quantified the released DNA to the supernatant as a global measure of neutrophil extracellular traps release (NETosis). Our data show that neither KPPR1 nor KP35 induced DNA release to the supernatant at 10 min (**Figure 3A**), and only after 3 h of infection, when most of KPPR1 has already been cleared, high concentrations of free DNA were found in KPPR1-infected neutrophils, but not in KP35-infected neutrophils (**Figure 3B**).

Altogether, these data show that KP phagocytic killing is the main mechanism used by neutrophils to rapidly clear KPPR1 *in vitro* and is uncoupled from cytokine production and NETs release. These data suggest differences in bacterial processing in neutrophils infected with KP35, which is able to evade neutrophil intracellular killing.

### Intracellular Response of Neutrophils to KP

**Figure 4:**
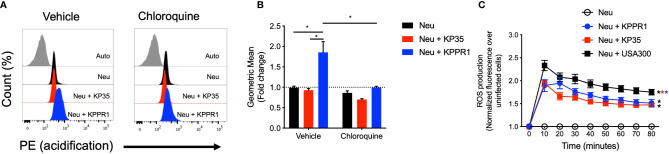
KP35 prevents neutrophil phagosome acidification but induces equivalent levels of ROS compared to KPPR1. **(A)** Isolated neutrophils were split in two groups and treated with chloroquine or vehicle. Then each group was infected with KPPR1 and KP35 and after 30 min **(B)** phagosome acidification was measured by flow cytometry. **(C)** ROS production by neutrophils was measured every 10 min for 80 min. *P < 0.05, each group was compared using a two-way ANOVA test followed by a Tukey’s test for multiple comparisons.

Two major events in neutrophil phagocytic killing are the increase of phagosome acidification and the production of reactive oxygen species (ROS) in response to phagocytosed bacteria. We measured phagosome acidification in neutrophils infected with KPPR1 or KP35 in the presence or absence of chloroquine, an inhibitor of lysosomal acidification (**Figures 4A, B**). Our data show that KPPR1-infected neutrophils had increased phagosome acidification that was significantly higher as compared to neutrophils infected with KP35, which did not present any change in phagosome acidification (**Figures 4A, B**).

Next, we evaluated reactive oxygen species (ROS) production by neutrophils in response to KPPR1 and KP35. Compared with uninfected neutrophils, KPPR1 and KP35 induced equivalent amounts of ROS *in vitro* (**Figure 4C**). This data shows that even though KP35 induces similar ROS production in neutrophils compared to KPPR1, KP35 impairs the processing and maturation of the bacterial phagosome required for KP intracellular killing.

### Effect of L-Arginine Supplementation in the Intracellular Killing of KP35 by Neutrophils

**Figure 5:**
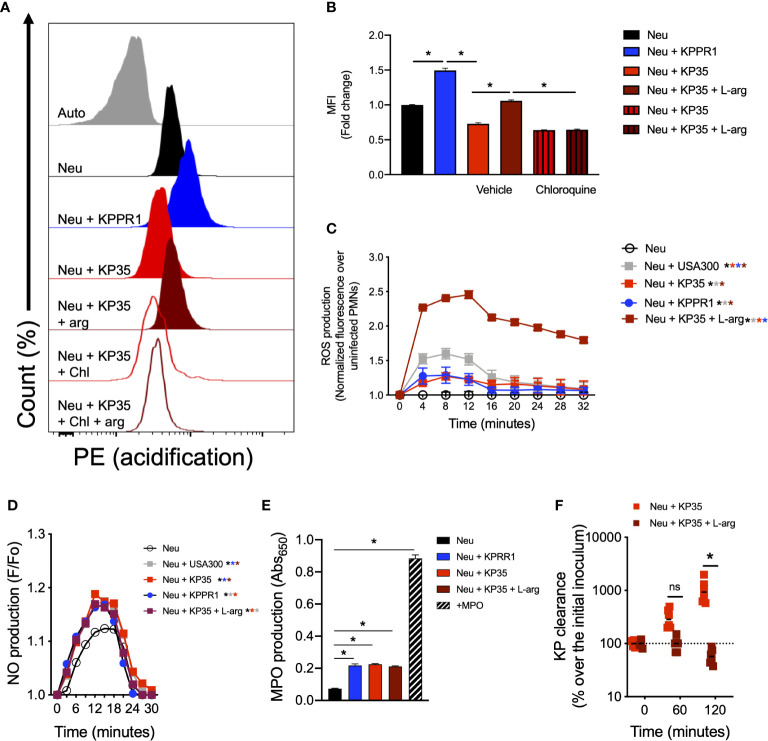
l-arginine improves phagosome acidification, ROS production, NO consumption and KP35 killing by murine neutrophils. Neutrophils were treated with 200mg/L of l-arginine and infected with KP35. **(A)** Phagosome acidification was evaluated and **(B)** quantified by flow cytometry after 30 min. **(C)** ROS production was measured every 4 min for 32 min. In parallel, **(D)** NO production was recorded every 3 min for 30 min. **(E)** MPO production was analyzed after 30 min. **(F)** KP35 killing was evaluated in presence or absence l-arginine for 2h. **(B, E)** *P < 0.05, each group was compared using a one-way ANOVA test followed by a Tukey’s test for multiple comparisons. **(C, D, F)** *P < 0.05, each group was compared using a two-way ANOVA test followed by a Tukey’s test for multiple comparisons. ns, non significant.

Next, we evaluated whether l-arginine supplementation could improve the neutrophil response against KP35. Our data show that l-arginine supplementation (200 mg/L) enhanced the neutrophil intracellular response against KP35. l-arginine supplementation improved phagosome acidification of neutrophils infected with KP35 after 30 min (**Figures 5A, B**), robustly improved the production of ROS (**Figure 5C**) and increased the consumption of nitric oxide (NO) during the first 30 min of infection (**Figure 5D**). However, it did not affect the neutrophil MPO activity, which was equivalently in response to KPPR1 and KP35 (**Figure 5E**). Ultimately, the improvement of the neutrophilic intracellular response followed by l-arginine supplementation, allowed these cells to efficiently kill KP35 after 2 h of infection (**Figure 5F**). In non-supplemented neutrophils KP35 proliferated about 1200% as compared to the initial inoculum. However, l-arginine-supplemented neutrophils killed 50% of KP35 (**Figure 5F**). To exclude the possibility that l-arginine directly affected KP35 growth, we tested the ability of KP35 to grow in LB broth (a rich-nutrient medium) and in HBSS (a poor-nutrient buffer) in the presence and absence of l-arginine. In both cases, l-arginine did not exert a deleterious effect in KP35 growth and even enhanced KP35 grow in HBSS (**Supplementary Figure 3**), supporting the conclusion that the increased KP35 clearance observed after l-arginine supplementation is due to enhanced neutrophil activity.

## Discussion

During classical KP infection, neutrophils drive the immune response and are major effectors in KP clearance and host survival; and their depletion leads to increased mortality and higher bacterial burden (Xiong et al., 2015). The central role of neutrophils described during KPPR1 pneumonia is different during CRKP-ST258 infections of the lung, where inflammatory monocytes recruitment is essential for bacterial clearance and host survival during both KPPR1 and CRKP-ST258 pneumonia (Xiong et al., 2015; Xiong et al., 2016), neutrophils seems to play a secondary role during CRKP-ST258 pulmonary infection. One previous report concluded that the evasion of neutrophil phagocytosis was the main mechanism by which CRKP-ST258 can survive killing by human neutrophils (Kobayashi et al., 2016). In this report we show that KP35, a clinical CRKP-ST258 isolate, does not prevent neutrophil phagocytosis but have the ability to evade intracellular killing. The main KP virulence factor that allows the evasion of neutrophil phagocytosis by KP is the polysaccharide capsule (Paczosa and Mecsas, 2016). Substantial heterogeneity in capsule composition and thickness among CRKP-ST258 isolates was recently described, ranging from hypomucoid to hypermucoid phenotypes (Ernst et al., 2020). Different mutations in the capsule biosynthesis genes located in the *cps* locus determines the capsule thickness (Ernst et al., 2020). Interestingly, the analysis of the *cps* locus showed that KP35 and KPPR1 lack *wbaP*, which is present in NJST258_1. On the other hand, KP35 and KPPR1 harbor in their *cps* loci the *wcaJ* gene, which is absent in NJST258_1. Both, *wbaP* and *wcaJ* encode the glycosyl-transferases responsible for the initiation of capsule biosynthesis; whereas *wbaP* encodes a galactose-1-P transferase, *wcaJ* encodes a glucose-1-P transferase instead (Rendueles, 2020), and the protein products of both of these genes have implications in the evasion of phagocytosis by KP (Pal et al., 2019; Ernst et al., 2020). Deletion of *wcaJ* promoted biofilm formation and allowed KP to evade macrophage phagocytosis and oxidative killing *in vitro* (Pal et al., 2019). Moreover, deletion of *wbaP* in a clinical isolate of CRKP-ST258 resulted in increased phagocytosis by macrophages *in vitro* (Ernst et al., 2020). We hypothesized that the presence of *wcaJ* in KPPR1 and KP35, as well as the differences observed in other glycosyl-transferase-encoding genes, renders these bacteria to be more susceptible to phagocytosis by neutrophils and macrophages, compared with NJST258_1, although more comprehensive studies are needed to corroborate this hypothesis.

Although KP35 is efficiently phagocytosed by neutrophils, our data show that neutrophils are unable to kill KP35. Our data is consistent with a recent report which shows that human neutrophils can efficiently phagocytose a CRKP-ST258 isolate, but have impaired microbicidal capacity due to an apparent deficiency in ROS production (Castillo et al., 2019). Our data show that *in vitro*, KP35-infected neutrophils produce similar ROS and MPO compared to KPPR1-infected neutrophils, suggesting that ROS and MPO inhibition are not mechanisms employed by KP35 to evade neutrophil killing but, instead may disturb other elements of the neutrophil phagosome machinery.

Moreover, CRKP-ST258 has been described to either impair or directly inhibit the release of NETs by human neutrophils (Castillo et al., 2019; Birnberg-Weiss et al., 2020). Our data are consistent with these findings and based upon the kinetics of KPPR1 killing and NETs production by KPPR-1 infected neutrophils, we hypothesized that the intracellular killing of KP by neutrophils is the main strategy employed by these cells to fight this bacterium. The evidence provided here and in other studies (Castillo et al., 2019) suggest that the mechanisms by which CRKP-ST258 evade neutrophil-phagocytic killing are not exclusive to phagocytosis but can also involve an active impairment of the intracellular killing machinery. A previous study has shown that KP has adapted to survive within macrophages phagosomes by preventing the phagosome-lysosome fusion and therefore the maturation of the phagolysosome (Cano et al., 2015). Although macrophages and neutrophil phagosomes are different, this is an important evidence that shows the ability of KP to adapt to these hostile environments.

The phagolysosome of macrophages and neutrophils work differently and the latter is characterized as being rapid and effective. ROS and HOCl production by NADPH oxidase and myeloperoxidase (MPO) respectively are major initiating events in neutrophil phagocytic killing (Hampton et al., 1998; Nordenfelt and Tapper, 2011). HOCl generation by MPO from O_2_^−^ and H^+^ pumped by the V-ATPase (Hampton et al., 1998; Nordenfelt and Tapper, 2011) results in the rapid alkalization of the phagosome (pH = 7.8), which is subsequently acidified by the action of a Na^+^/H^+^ antiport and the V-ATPase, reaching a pH = 6 (Hampton et al., 1998). These changes in pH in the neutrophil phagosome are important to allow the optimal function of the different proteins involved in pathogen clearance and highlights how phagosome acidification is a good indicator of phagosome maturation in neutrophils. MPO optimally functions at pH = 6, whereas the optimal pH of proteases such as Elastase or Cathepsin G is around 7 and 10, respectively (Levine et al., 2015). The importance of phagosome acidification is seen with *S. pyogenes* infection, which prevents phagosome acidification through the impairment of the V-ATPase activity, leading to prolonged survival inside the neutrophil (Nordenfelt et al., 2012). Our data show that after the phagocytosis of KPPR1, the intracellular response that promotes ROS and MPO production, along with rapid phagosome acidification, leads to highly efficient killing of KPPR1. However, despite the equivalent ROS and MPO production observed in KP35-infected neutrophils, neutrophils fail to acidify their phagosome and ultimately are not able to kill KP35, again suggesting a disruption in phagosome maturation. This conclusion is consistent with previous data showing that neutrophils infected with KP35 fail to induce Ca^2+^ flux in the cytoplasm (Ahn et al., 2016), an important event in actin-polymerization and phagosome maturation (Nordenfelt and Tapper, 2011) and given that MPO activity is optimized at acidic pH, it is possible that MPO activity in the KP35-infected neutrophil is reduced compared to KPPR1-infected neutrophils. How KP35 disrupts neutrophil activity and which virulence factors are involved in this process is unknown, however, a type VI secretion system has been recently identified in KP with a potential role in cell invasion (Hsieh et al., 2019; Storey et al., 2020) and the possibility that injected virulence factors alters the normal function of the neutrophil intracellular killing machinery is an attractive hypothesis.

Despite evasion of neutrophilic killing of CRKP-ST258 through different mechanisms, neutrophils still have an important role in CRKP-ST258 clearance *in vivo*. Neutrophil depletion impairs lung CRKP-ST258 clearance (Ahn et al., 2016) and impaired neutrophil activity can be associated to impaired CRKP-ST258 clearance (Peñaloza et al., 2019b), indicating that *in vivo*, neutrophils are important cells in CRKP-ST258 clearance. Indeed, CRKP-ST258 phagocytic killing by neutrophils can be improved after antibody-mediated opsonization directed against the polysaccharide capsule *in vitro* (Kobayashi et al., 2018).

Moreover, neutrophils can interact with several immune and non-immune cells (Mantovani et al., 2011) and local microenvironment shapes neutrophil function and activity (Mayadas et al., 2014). l-arginine has been recognized as an important immunomodulator in the lungs (Mabalirajan et al., 2010) and even though is controversial, some studies show that l-arginine nutritional support may have an effect in the prevention of nosocomial infections in the ICU and in the survival of septic ICU patients (Bower et al., 1995; Galban et al., 2000; Caparros et al., 2001; Bertolini et al., 2003; Kieft et al., 2005),

Particularly in neutrophils, l-arginine has positive effects in the neutrophilic response (Canturk et al., 2001; Chen et al., 2015), including phagocytosis (Moffat et al., 1996). In our study, we demonstrated that l-arginine supplementation at a concentration present in RPMI medium (200 mg/L) was enough to improve KP35 killing after 2 h and to increase phagosome acidification, ROS production and NO consumption during the first 30 min. l-arginine is the substrate of inducible nitric oxide synthase (iNOS), which catalyzes the production of nitric oxide (NO), an important molecule involved in the respiratory burst and an active substrate of reactive nitrogen species (RNS), important antimicrobial factors produced during phagocytic killing in neutrophils (Saini and Singh, 2019). RNS and ROS production are intimately related, as NO and O^2-^ are the substrates of peroxynitrite (ONOO^−^) which is a highly antimicrobial molecule (Uribe-Querol and Rosales, 2017). We speculate that the increased NO consumption observed in l-arginine supplemented neutrophils infected with KP35 may be due to the increased formation of ONOO^-^. Alternatively, it is possible that the enhanced killing of KP35 by l-arginine supplemented neutrophils is due to increased ROS production. Neutrophils can metabolize l-arginine (Muhling et al., 2002), and a previous report shows that after phagocytosis, iNOS, the enzyme that catalyzes the production of NO from the substrate l-arginine, can interact with Rac2 and form a complex that is critical for ROS, RNS, NO and superoxide production in the neutrophil phagosome (Jyoti et al., 2014).

Whereas a strong body of evidence has shown the ability of KP to evade neutrophil phagocytosis, the data presented in this report and in previous recent studies conclude that some isolates of CRKP-ST258 have acquired the ability to survive in the intracellular environment of these cells. Whether l-arginine supplementation improves the neutrophil-mediated killing on CRKP-ST258 isolates that evade phagocytosis is unknown and should be evaluated; however, the diversity of evasion strategies used by CRKP-ST258 continue to challenge scientists and physicians in the therapeutic approach to fighting these infections from multiple angles, pushing the design of different therapeutic approaches to combat unique strategies used by KP to evade the neutrophil phagocytic response.

## Conclusions

In this report we provide evidence that the evasion of neutrophil killing by CRKP-ST258 is not restricted to the evasion of phagocytosis, as previously reported, but also due to the impairment of intracellular killing machinery. This neutrophilic resistance can be reversed after l-arginine supplementation. These findings have enormous potential, since they open the door for new therapeutic alternatives that may improve the immunity against pathogenic bacteria resistant to several antibiotics. Despite these promising insights into how l-arginine boosts the intracellular killing machinery in neutrophils, more studies are required to deeply understand the molecular effects of l-arginine supplementation in the neutrophil biology against pathogenic bacteria and the consequences of l-arginine supplementation *in vivo*.

## Data Availability Statement

The raw data supporting the conclusions of this article will be made available by the authors, without undue reservation.

## Ethics Statement

The animal study was reviewed and approved by Comité Etico Científico en Cuidado Animal y Ambiente. Pontificia Universidad Católica de Chile.

## Author Contributions

All authors contributed to the article and approved the submitted version.

## Funding

This study was supported by grants from Fondo Nacional de Desarrollo Científico y Tecnológico de Chile (grant no. 1170964), the Millennium Institute on Immunology and Immunotherapy (ICN09_016), the Comisión Nacional de Investigación Científica y Tecnológica de Chile (grant no. 21140214), and NIH K08 HL138289.

## Conflict of Interest

The authors declare that the research was conducted in the absence of any commercial or financial relationships that could be construed as a potential conflict of interest.
