# Supplementary material for: L-Arginine Enhances Intracellular Killing of Carbapenem-Resistant Klebsiella pneumoniae ST258 by Murine Neutrophils
Source: Front Cell Infect Microbiol. 2020 Nov 13;10:571771. doi: 10.3389/fcimb.2020.571771 (PMC7691228; doi:10.3389/fcimb.2020.571771)
Supplement: Supplementary file 1 [file DataSheet_1.pdf]

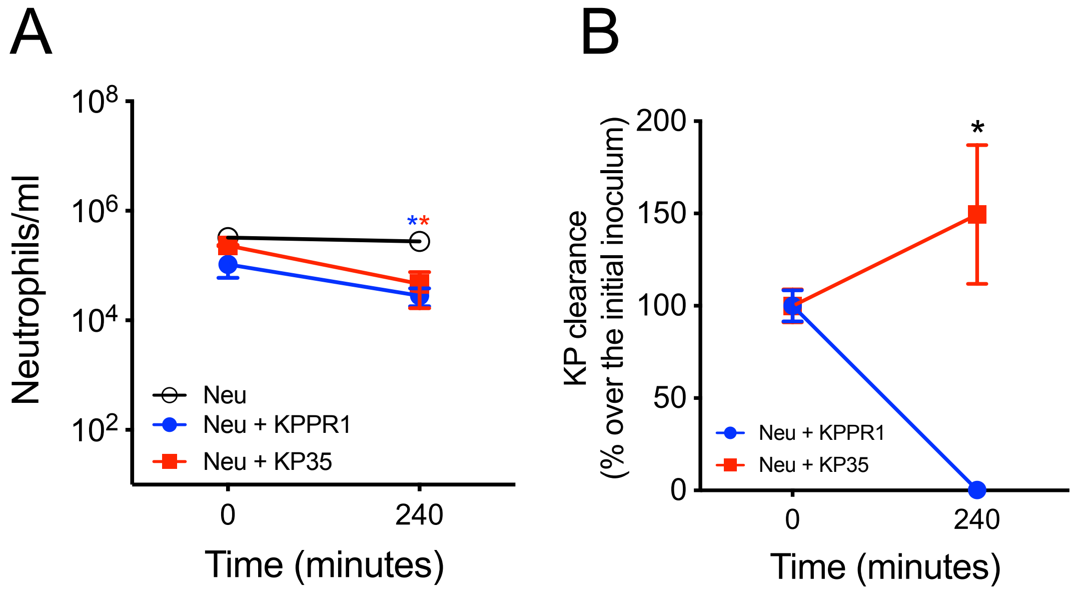

Supplementary Figure 1: Neutrophil survival and KP killing at 4 h. (A) KP35 and KPPR1 infected neutrophils count and (B) bacterial killing (MOI=1) were evaluated for 4h. * $P<0.05$, each group was compared using a Two-way ANOVA test followed by a Tukey's multiple test comparison.

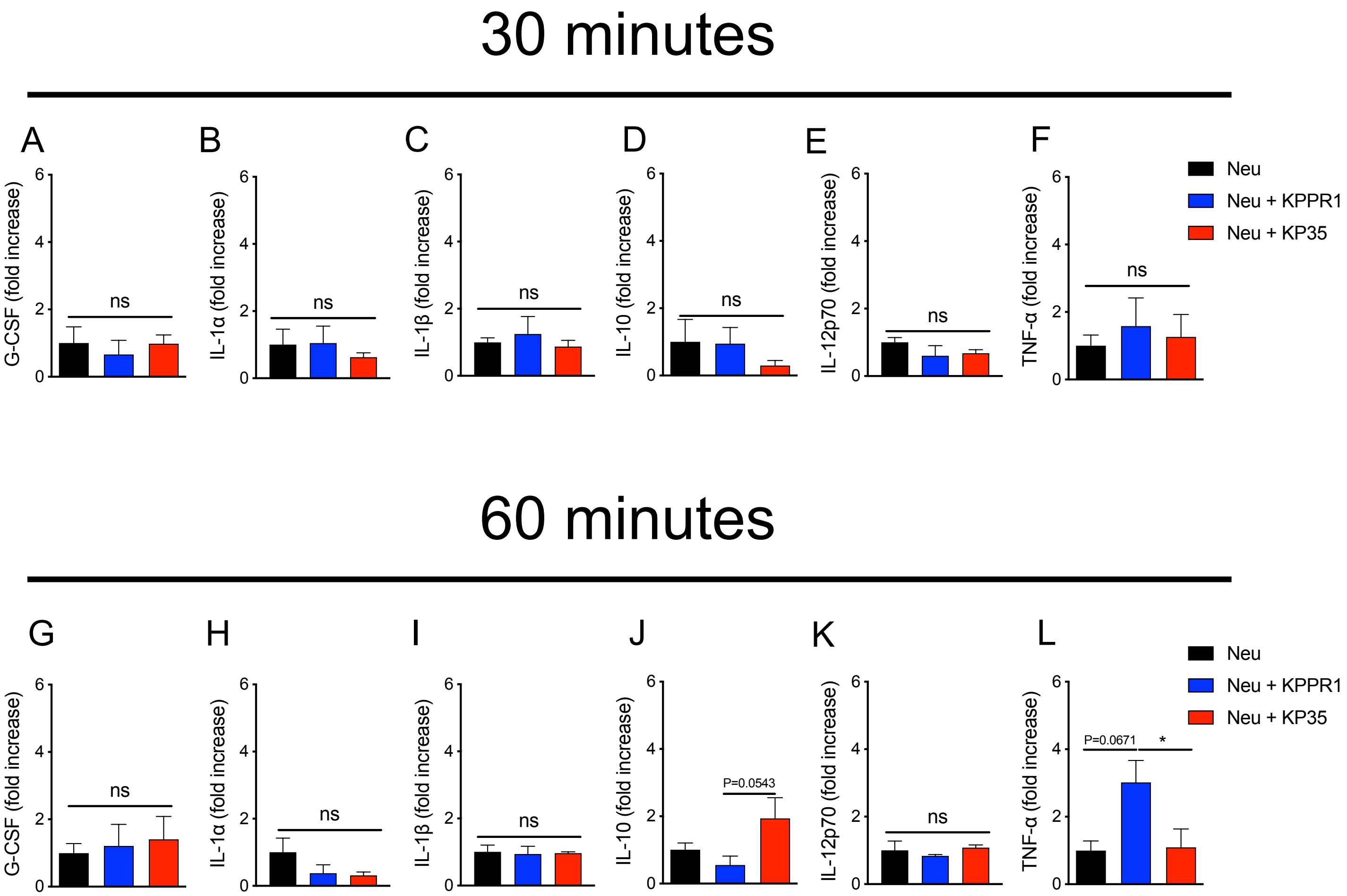

Supplementary Figure 2: Cytokine production by neutrophils during KP infection. Production of (A-F) G-CSF, IL-1α, IL-1β, IL-10, IL-12p70 and TNF-α at 30 min and (G-L) 60 min after KP35 and KPPR1 infection. For each analyte, data was normalized over uninfected neutrophils. * $P<0.05$, each group was compared using a One-way ANOVA test followed by a Tukey's multiple test comparison.

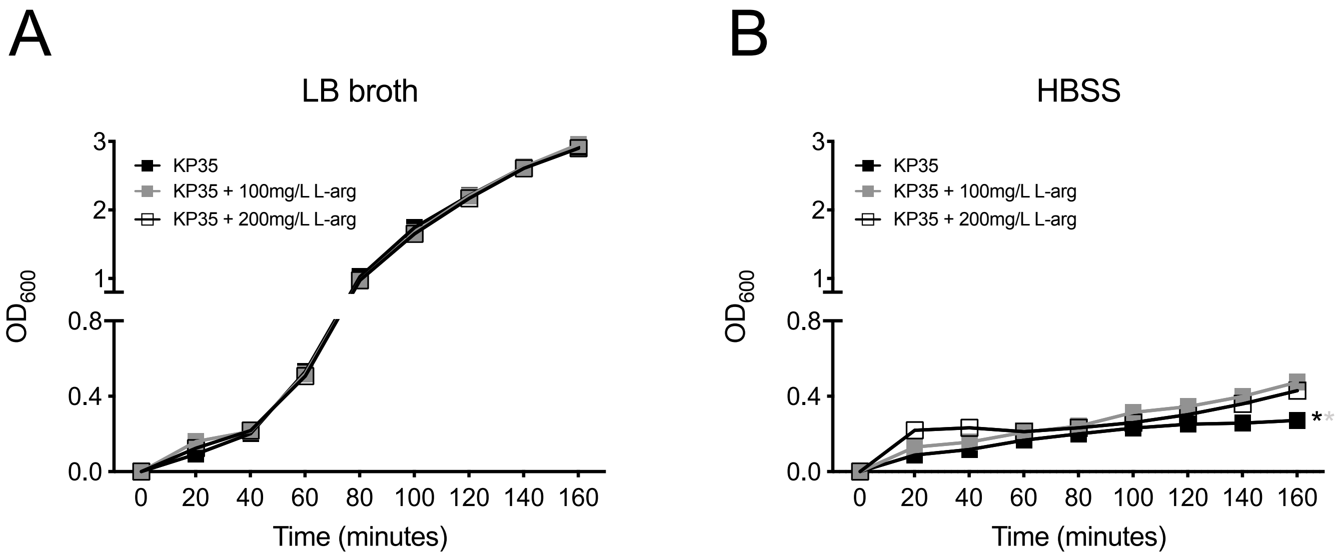

Supplementary Figure 3: L-arginine supplementation improves KP35 growing in a (A) rich and (B) poor nutrient media. * $P<0.05$, each group was compared using a Two-way ANOVA test followed by a Tukey's multiple test comparison.
